# Necrotizing Ulcerative Gingivitis, a Rare Manifestation as a Sequel of Drug-Induced Gingival Overgrowth: A Case Report

**DOI:** 10.1155/2021/4120148

**Published:** 2021-09-21

**Authors:** Marah Damdoum, Sudhir Rama Varma, Mohamed A. Jaber, Manjusha Nambiar

**Affiliations:** ^1^Department of Clinical Sciences, College of Dentistry, Ajman University, Ajman, UAE; ^2^Centre of Medical and Biomedical Allied Health Sciences Research, Ajman University, Ajman, UAE; ^3^Department of Periodontics, Sri Rajiv Gandhi College of Dental Sciences and Hospital, Banguluru, Karnataka State 560032, India

## Abstract

**Purpose:**

The purpose of this case report is to present a rare case of amlodipine-induced gingival overgrowth with a secondary formation of necrotizing ulcerative gingivitis involving the upper and lower arches of a 68-year-old female patient with a chief complaint of “swollen gums and pain on mastication which has been recurring for the past 5 years.”

**Materials and Methods:**

The treatment plan of this case was divided according to quadrants of the mouth. Each week, one quadrant was surgically excised, and the remaining quadrants were observed for any changes. The gingival overgrowths were excised using a 15 blade, and debris/plaque was removed with Gracey curettes.

**Results:**

Although full-mouth exodontia was performed, the patient unfortunately suffered with recurrences in GO. These results are suggestive of idiopathic causes of GO.

**Conclusion:**

Careful examination, physician referrals, and biopsy to rule out any specific anomalies and to assist in proper diagnosis are followed by sequential management of the case results in productive outcomes.

## 1. Introduction

Drug-induced gingival overgrowth (DIGO) manifests as a result of drug interactions mainly produced by anticonvulsants [1], immunosuppressants [2], and calcium channel blockers [3]. Several factors including age, sex, genetic predisposition, systemic diseases, hormonal imbalances, congenital disorders, poor oral hygiene, and local plaque/debris may influence the relationship of these drugs to gingival overgrowth [4]. Gingival overgrowths can affect speech, mastication, and esthetics, and there are various methods of treatment for excising the excess tissue [5]. These overgrowths can be classified according to their size and location and graded between grade 0 and grade III [6] (Table 1). The pharmacological action of each drug is different; however, they all act similarly to secondary tissue receptors by creating enlargements in the gingival tissue. The exact mechanism of these gingival overgrowths in association to drugs is not fully understood, and there are many hypotheses and theories related to DIGO [7–11]. Drug-induced gingival overgrowth is typically detected after 3 months of drug consumption [12]. Clinical features of DIGO could be localized or generalized and may range from mild to severe enlargements of the marginal and papillary gingiva [13]. The anterior region of the mouth is more affected than the posterior region and is more pronounced on the facial surfaces than the palatal/lingual surfaces [14]. DIGO cannot be prevented in susceptible patients but it can be tolerated by regular periodontal therapy and plaque control [15]. The current case report presents a rare case of necrotizing ulcerative gingivitis as a secondary reaction to amlodipine-induced gingival overgrowth.

## 2. Pathophysiology

The mechanism of gingival overgrowth and its contributory mechanism with relation to fibroblastic proliferation are varied. A theory of genetic predisposition was earlier postulated by Seymour et al. in 1996 [3]. The inflammatory changes and responses of gingival fibroblasts coupled with connective tissue matrix proliferation exemplify the fibroblastic characterization with relation to the various drugs responsible for this change [16]. Another theory postulated that bacterial plaque was responsible for gingival inflammation. The drugs responsible for gingival overgrowth causes an increased production of glycosaminoglycans. The drugs further reduce folate uptake in the cellular matrix. Reduced folate uptake results in decreased synthesis of matrix metalloproteinases which is responsible for conversion of collagenase precursor into mature collagenase, resulting in a matrix and connective tissue build up. The bacterial plaque when present causes the inflammatory component, thus completing the cycle [17].

## 3. Case Report

This case presents a 68-year-old female patient with a chief complaint of swollen gums and pain on mastication which has been recurring for the past 5 years. According to the patient, she started taking amlodipine 5 mg/day 7 years ago and started experiencing enlargements of the gingiva 5 years ago. She stopped taking amlodipine for 6 months and claims the enlargements improved. However, her physician recommended that she continue taking amlodipine 10 mg/day due to her uncontrolled hypertension. Medical history was taken in which the patient reported having diabetes mellitus type II and hypercholesterolemia for the past 8 years. She takes a series of drugs for her medical conditions (Table 2). She has been hypertensive for the past 7 years and has been taking amlodipine since then. She stopped amlodipine 2 years ago for 6 months due to gingival overgrowths; however, amlodipine had to be continued due to her uncontrolled hypertension.

On taking dental history, it was found that she underwent multiple gingivectomies in the past with recurrence every few months. During previous episodes, the patient had described the condition as similar to the one presented currently with excruciating pain and pus discharge. Furthermore, the patient described the treatment advised as “gum shaping by the doctor”. From patient's explanations, it was assumed that the clinical picture matched the current situation, and a history of NUG could have been prevalent at that time period.

**Figure 1:**
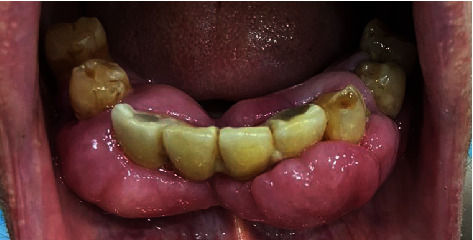
Fibrous, nodular, hard gingival overgrowths extending to most of the clinical crown in the anterior and posterior parts of the mandibular arch.

**Figure 2:**
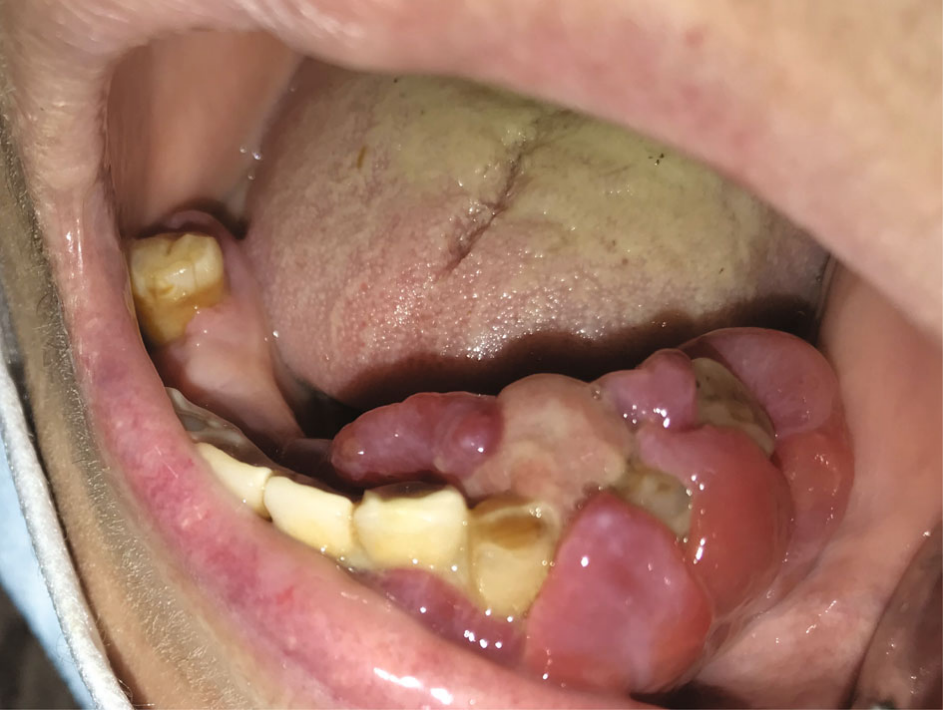
Yellowish-white pseudomembranous slough was found covering the interdental papilla in the mandibular left area.

**Figure 3:**
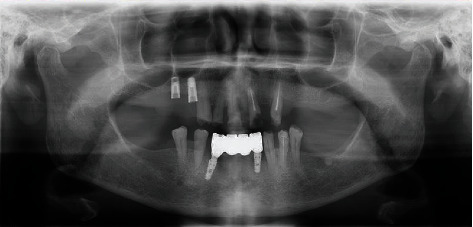
Panoramic radiograph presenting bone loss around teeth and lower 4-unit implant-retained bridge.

The patient presented with a dental history that includes thick deposits of calculus and plaque on all remaining teeth with fibrous, nodular, hard gingival overgrowths extending to most of the clinical crown in the anterior and posterior parts of the maxillary and mandibular arches (Figure 1). The overgrowths can be classified as grade III according to Bokenkamp's 1994 classification for gingival overgrowth [6] (Table 1). Additionally, a yellowish-white pseudomembranous slough was found covering the interdental papilla in the mandibular left area (Figure 2). Radiographically, bone loss can be observed around the teeth and implants; however, clinically, the implants are not mobile as submerged (Figure 3). Abrasion of the teeth can be observed indicating parafunctional habits, as the patient had given a history of clenching which was consciously done and also from her family member, that patient was a bruxer and exhibited grinding at night. This also confirmed our diagnosis as stage 4, grade C periodontitis, currently unstable.

### 3.1. Sequence of Treatment

**Figure 4:**
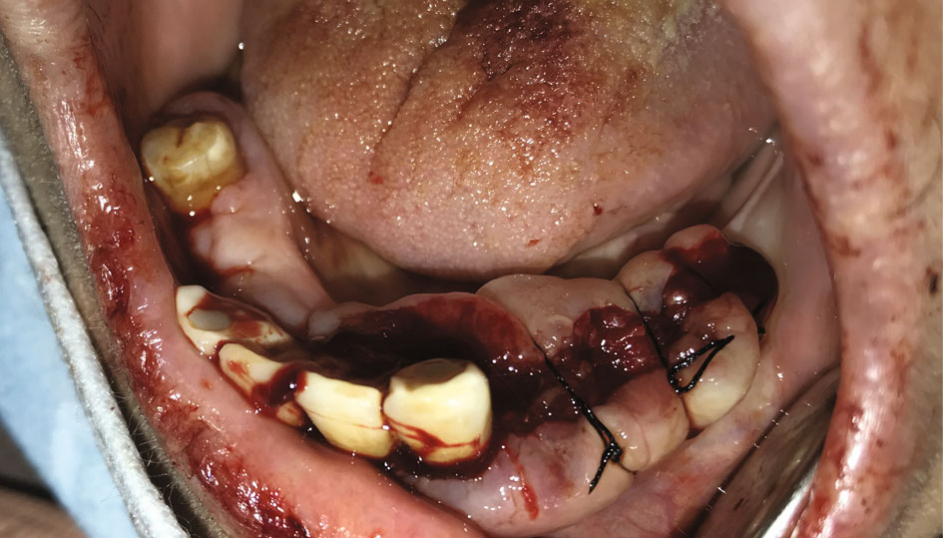
External bevel incision gingivectomy of the lower left quadrant and exodontia of #33, #34, and #35.

**Figure 5:**
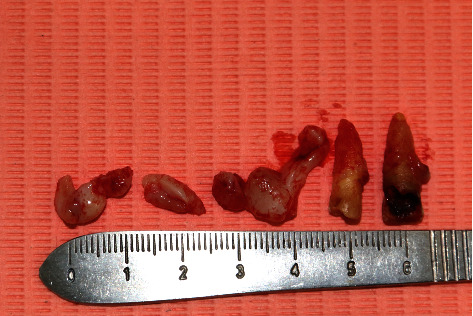
Gingiva removed from the lower left quadrant and extracted teeth (#33, #34, and #35).

**Figure 6:**
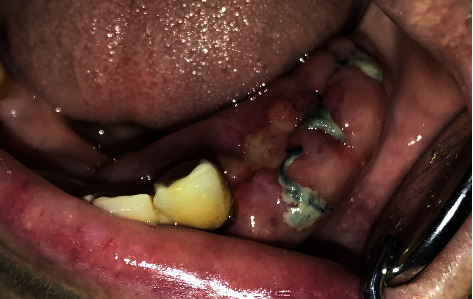
1 week after surgical excision of the lower left quadrant.

**Figure 7:**
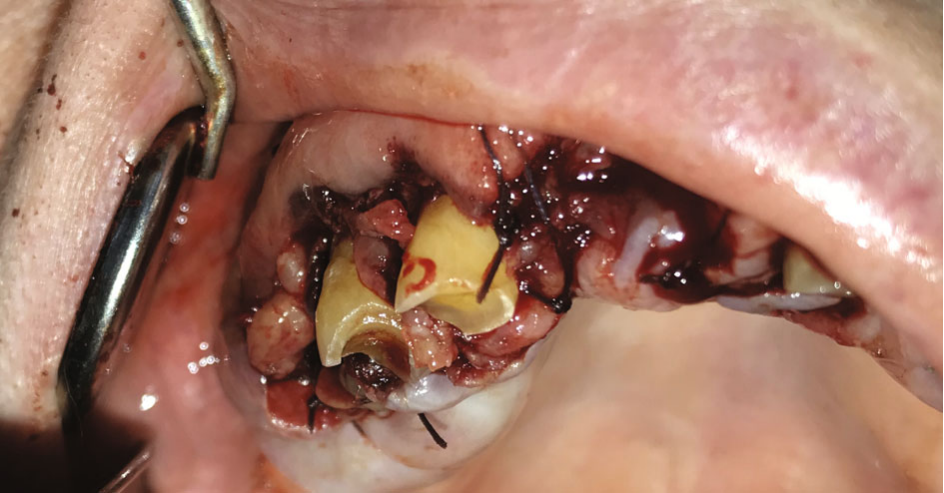
External bevel gingivectomy of the upper right quadrant.

**Figure 8:**
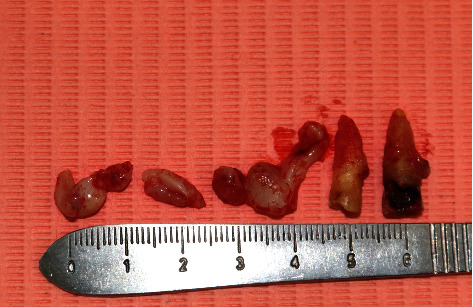
Gingiva removed from the upper left quadrant and extracted teeth (#11 and #21).

**Figure 9:**
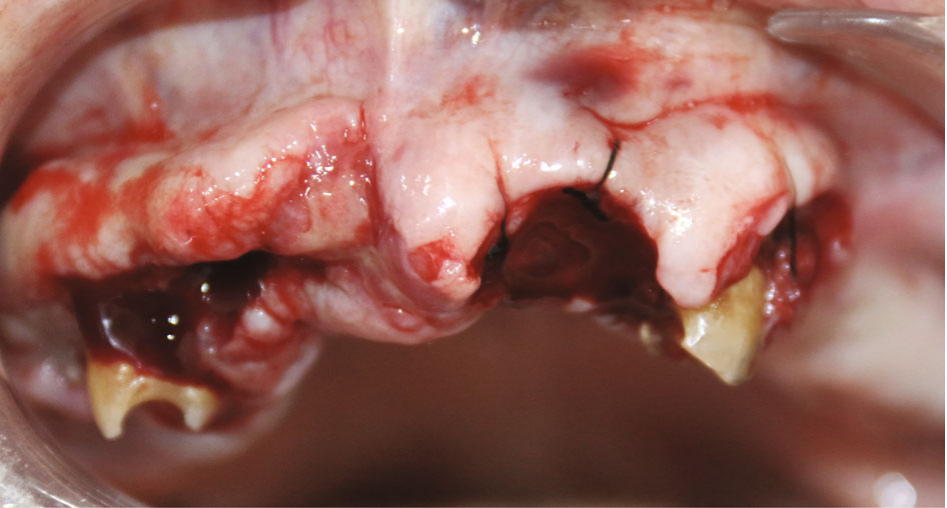
External bevel gingivectomy of the upper left quadrant.

The treatment plan of this case was divided according to quadrants of the mouth. Each week, one quadrant was surgically treated with the overgrown gingival tissue being excised, and the mobile teeth were extracted. The decision to extract her teeth was from a multidisciplinary point of view involving a periodontist and prosthodontist, as salvaging the remaining teeth was challenging and time consuming with hopeless prognosis. Since the overgrowths affected mastication, speech, and esthetics, a surgical approach (i.e., gingivectomy) was utilized with local anesthesia being administered. The gingival overgrowths were excised using a 15 blade; plaque and calculus were removed with Gracey curettes (Dentsply, USA). After every gingivectomy, azithromycin-xithrone (Amoun Egypt) 500 mg, diclofenac-rofenac (Bin Sina, UAE) 50 g sachet, along with chlorhexidine mouthwash (Curasept, UK), were prescribed. Surgical excision began in quadrant 4, which was the least affected quadrant. An external bevel incision gingivectomy was performed along with scaling and root surface debridement. The gingiva was sutured with silk (Black Braided, size 4-0, 19 cm, Teleflex, USA). At one-week surgical postop, the following was noted: the gingiva in the mandibular right quadrant was healing within normal limits. The gingiva in the mandibular left quadrant was edematous, fibrous, hemorrhagic, and painful swellings which had purulent exudate (Figure 2). An external bevel incision gingivectomy was done along with extraction of #33, #34, #35, and 45 due to extensive grade III mobility (Figures 4 and 5). The mobile teeth were diagnosed as stage 4 grade C periodontitis, currently unstable (AAP Classification, 2017). Apical curettage was performed, and copious irrigation with saline was done. Simple interrupted sutures were placed with silk. Placement of a periodontal pack along with oral hygiene reinforcement instructions was given. During the follow-up visit the following week, healing was uneventful for the most part with slight regression of gingival overgrowth. Poor oral hygiene can be observed along the sutures which were surrounded by plaque and debris (Figure 6). For the maxillary arch, an external bevel incision gingivectomy was done, and extraction of #11, #21, and #12 was done due to grade III mobility (Figures 7–9).

**Figure 10:**
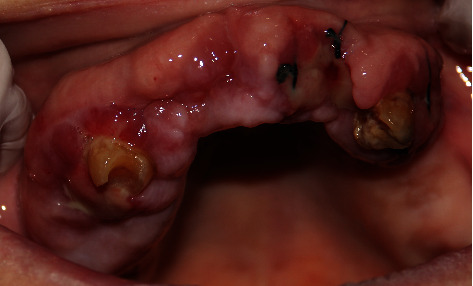
Recall after 2 weeks of surgical intervention of maxillary arch. Pus and exudates were observed around teeth and sutures. Full exodontia was done for the remaining teeth in the upper arch.

**Figure 11:**
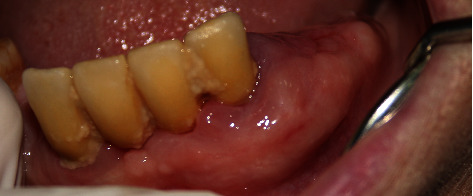
1 month recall after surgical intervention of the mandibular arch. Recurrence was presented, and full-mouth exodontia was done for the lower arch.

During the follow-up visits, the maxillary arch presented with signs of inflammation, pus, and exudates around the teeth and sutures (Figure 10). There also seemed to be recurrence of gingival overgrowth around the implant-retained 4 unit bridge in the anterior mandible (Figure 11). The bridge was removed, and implants remained submerged in the alveolar bone. After bridge removal, azithromycin was prescribed as reported in earlier literature about its efficiency in treating DIGO [18]. During a 2-week follow-up, recurrence of GO was identified in all quadrants. The patient was referred to a cardiologist for a follow-up and possible substitution of amlodipine. Perindopril indapamide (a thiazide-like diuretic) was recommended by the cardiologist as an alternative. The remaining drugs consumed by the patient continued normally. During the first recall visit after complete exodontia and drug substitution, the patient presented with recurrence in the upper and lower arches. Since gingival overgrowth did not subside after complete exodontia and drug substitution, it can be assumed that the submerged implants in the lower anterior region and upper right posterior region could be the cause for this recurrence. Treatment modalities were further contemplated with regard to removal of the submerged implants; however, after clinical and radiographic assessment, it was further decided to retain the implants and to put the patient on follow-up to see if there was a possibility of recurrence. The patient was put on a strict oral hygiene plan and maintenance recall phase every 2 months.

## 4. Results

This case report presents a rare case of nonhealing AIGO with a secondary NUG reaction. Two biopsies were taken to confirm the diagnosis in which both biopsies revealed chronic inflammatory processes. For AIGO-associated conditions, there are a range of treatment modalities that clinicians can explore. The proposed modes of treatment in the literature include nonsurgical and surgical drug modification and periodontal therapies. For the current case, all of these modalities were performed, and biopsies were done to confirm NUG as a manifestation of DIGO. Although full-mouth exodontia was performed, the patient unfortunately suffered with recurrences in GO. These results are suggestive of idiopathic causes of GO.

## 5. Discussion

### 5.1. Oral Hygiene

Some studies say oral hygiene and local factors like plaque and calculus can affect the severity of gingival overgrowth; however, it is not a requirement for DIGO [4, 19]. A crosssectional study done in 1973 tested the association between dilantin-induced gingival overgrowth and oral hygiene. The study did not show that oral hygiene was statistically significant in the formation of gingival hyperplasia [4]. Seymour et al. reported in 2000 that most of the evidence to support a relationship between bacterial plaque and gingival overgrowth has been derived from crosssectional studies, and it is not clear whether plaque is a contributory factor or a consequence of the gingival changes [4]. A 2015 case report by Mathur et al. took a nonsurgical approach to their DIGO case. They reported that “there appears to be three significant factors which are important for the expression of these gingival changes, notably drug variables, plaque-induced inflammatory changes in gingival tissue, and genetic factors which determine the heterogeneity of the fibroblasts” [19]. The gingival overgrowths subsided after periodontal therapy, and no surgical intervention was needed. In a 2020 case report by Uppal et al., they report a case of DIGO that was treated nonsurgically through a periodontal approach first and report that “as the enlargement did not subside after drug substitution and phase 1 therapy, surgical phase was planned further” [20]. There is definitely an unclear definition as to whether oral hygiene plays a role in DIGO, but regardless, patient education on oral hygiene and maintenance is crucial in every treatment plan.

### 5.2. Histopathology

For this case, a biopsy was sent with a gross description of the tissue sample; it consisted of two pieces of gray white to brown tissue measuring 2.0 × 0.8 × 0.5 cm and 1.5 × 1.4 × 0.8 cm.

Microscopically, the histopathological sections show mucosa covered soft tissue with an area of ulceration along with granulation tissue. Subepithelial soft tissue is hyalinized and shows a dense infiltrate composed predominantly of plasma cells merged with a few lymphocytes, eosinophils, and neutrophils. These features are consistent with acute on chronic inflammatory processes. The predominance of inflammatory cells indicates that the gingival overgrowth was not only caused by a drug induction but rather by presence of a secondary condition as well. This secondary condition can be identified as necrotizing ulcerative gingivitis. In the literature, the histopathology of NUG has been identified as ulcerations with dense infiltrations of PMNs and plasma cells which is interpreted as an area of established chronic gingivitis on which the acute lesion became superimposed [2].

**Figure 12:**
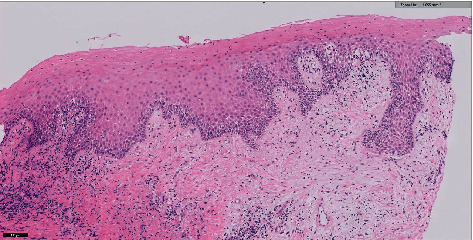
Histopathological slide of gingival biopsy revealed squamous epithelium hyperplasia with underlying chronic inflammation.

**Figure 13:**
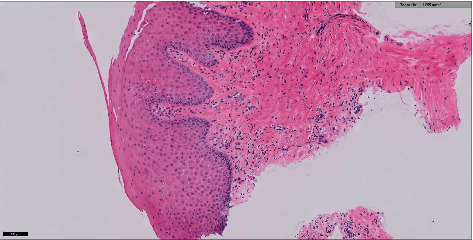
Histopathological slide of gingival biopsy revealed squamous epithelium hyperplasia with underlying chronic inflammation.

Our biopsy showed areas of ulcerations, predominance of plasma cells (indicating a chronic lesion), along with some PMNs. The signs and symptoms along with the histopathological review were suggestive of NUG as a secondary reaction to AIGO. A second biopsy was taken of healthy gingival tissue, to confirm our diagnosis of AIGO. The biopsy was received in a labelled container with patient identification and fixed in formalin, where multiple pieces are gray brown in color, irregular soft tissue, and measured in aggregate 1.5 × 1 × 0.2 cm. All submitted in one cassette. The gingival biopsy revealed squamous epithelium hyperplasia with underlying chronic inflammation (Figures 12 and 13), consistent with a reactive process, and negative for hyperkeratosis and atypia. These reports are consistent with a secondary inflammatory reaction (NUG) as a manifestation of amlodipine-induced gingival overgrowth [21]. The current case is similar to a case report where DM type 2, hypercholesterolemia, and amlodipine for hypertension were of similar findings. The difference being the time frame for the presentation, in the current case, was nine months ago, while in the earlier reported literature, it had presented after three years of intake of the medication (amlodipine) [22]. Meanwhile, our patient has had GO for the past 5 years and experienced the changes in her gingiva almost instantly after switching back to amlodipine the second time. Interestingly, in the current case, a possible role of statins in the progression of GO could be attributed as seen from a previous study where statins and calcium channel blockers when prescribed together resulted in adverse effects, a predominant feature being GO [23].

### 5.3. Secondary Reactions

In our case, we reported necrotizing ulcerative gingivitis (NUG) as a secondary reaction to amlodipine-induced gingival overgrowth. In an earlier study by Vishnusdas et al., they reported a case involving amlodipine-induced gingival plasma cell granuloma, and the etiology is multifactorial, with a possible role of drug/cellular interaction playing a contributory role in the pathogenesis of this entity [24]. Another case report done in 2019 by Gulati et. al found an unusual plasma cell granuloma formation secondary to GO. This case report states that biopsies for unusual lesions should be comprehensively examined regardless of clinical appearance or treatment success rate in order to rule out neoplasms and plan for treatment accordingly [21]. For this case, a biopsy was sent with a gross description of the tissue sample, it consisted of two pieces of gray white to brown tissue measuring 2.0 × 0.8 × 0.5 cm and 1.5 × 1.4 × 0.8 cm. Microscopically, the histopathological sections show mucosa covered soft tissue with an area of ulceration along with granulation tissue. Subepithelial soft tissue is hyalinized and shows a dense infiltrate composed predominantly of plasma cells merged with a few lymphocytes, eosinophils, and neutrophils. These features are consistent with acute on chronic inflammatory processes. The predominance of inflammatory cells indicates that the gingival overgrowth was not only caused by a drug induction, but rather by presence of a secondary condition as well. This secondary condition can be identified as necrotizing ulcerative gingivitis. In the literature, the histopathology of NUG has been identified as ulcerations with dense infiltrations of PMNs and plasma cells which is interpreted as an area of established chronic gingivitis on which the acute lesion became superimposed [2]. Our biopsy showed areas of ulcerations, predominance of plasma cells (indicating a chronic lesion), along with some PMNs. The signs and symptoms along with the histopathological review were suggestive of NUG as a secondary reaction to AIGO. A second biopsy was taken of healthy gingival tissue, to confirm our diagnosis of AIGO. The biopsy was received in a labelled container with patient identification and fixed in formalin, where multiple pieces are gray brown in color, irregular soft tissue, and measured in aggregate 1.5 × 1 × 0.2 cm. All submitted in one cassette. The gingival biopsy revealed squamous epithelium hyperplasia with underlying chronic inflammation (Figures 12 and 13). Consistent with a reactive process. Negative for hyperkeratosis and atypia. These reports are consistent with a secondary inflammatory reaction (NUG) as a manifestation of amlodipine-induced gingival overgrowth.

The current case is similar to a case report where DM type 2, hypercholesterolemia, and amlodipine for hypertension were of similar findings. The difference being the time frame for the presentation, in the current case, was nine months ago, while in the earlier reported literature, it had presented after three years of intake of the medication (amlodipine) [22]. Meanwhile, our patient has had GO for the past 5 years and experienced the changes in her gingiva almost instantly after switching back to amlodipine the second time. Interestingly, in the current case, a possible role of statins in the progression of GO could be attributed as seen from a previous study where statins and calcium channel blockers when prescribed together resulted in adverse effects, a predominant feature being GO [23].

## 6. Conclusion

DIGO related to calcium channel blockers, immunosuppressants, and anticonvulsants in the past reported clinical presentations concluded by clinicians as rare entities. The case presented in our study shows the possible effects of amlodipine-induced GO and the challenges dentists can face in the management of this condition. Careful examination, physician referrals, and biopsy to rule out any specific anomalies and to assist in proper diagnosis are followed by sequential management of the case results in productive outcomes.

## Figures and Tables

**Table 1:** Bokenkamp and Bohnhorst, 1994 classification.

Grade	Clinical findings
Grade 0	No signs of gingival overgrowth
Grade I	Gingival hyperplasia confined to interdental papilla
Grade II	Hyperplasia of interdental papilla and marginal gingiva
Grade III	Gingival hyperplasia covering at least three quarters of tooth crowns

**Table 2:** Drugs taken by the patient.

Name of the drug	Dosage	Duration	Pharmacological action	Medical condition
Gliclazide	60 mg	8 years	Sulfonylureas	Diabetes mellitus type II
Bisoprolol hemifumarate	2.5 mg	1 year	Nonselective beta blocker	Heart murmurs
Perindopril arginine	5 mg	1 year	ACE inhibitor	Hypertension
Amlodipine besylate	10 mg	7 years	Calcium channel blocker	Hypertension
Rosuvastatin	10 mg	8 years	HMG-CoA reductase inhibitor	Hypercholesterolemia

## Abbreviations

DIGO: Drug-induced gingival overgrowth
AIGO: Amlodipine-induced gingival overgrowth
GO: Gingival overgrowth
NUG: Necrotizing ulcerative gingivitis
PMN: Polymorphonuclear cells.
